# Supplementary material for: Spatiotemporal development of late and moderate preterm infant gut and oral microbiomes and impact of gestational age on early colonization
Source: mSystems. 2025 Nov 24;10(12):e00667-25. doi: 10.1128/msystems.00667-25 (PMC12710329; doi:10.1128/msystems.00667-25)
Supplement: Supplemental figures — Figures S1 to S9. [file msystems.00667-25-s0001.pdf]

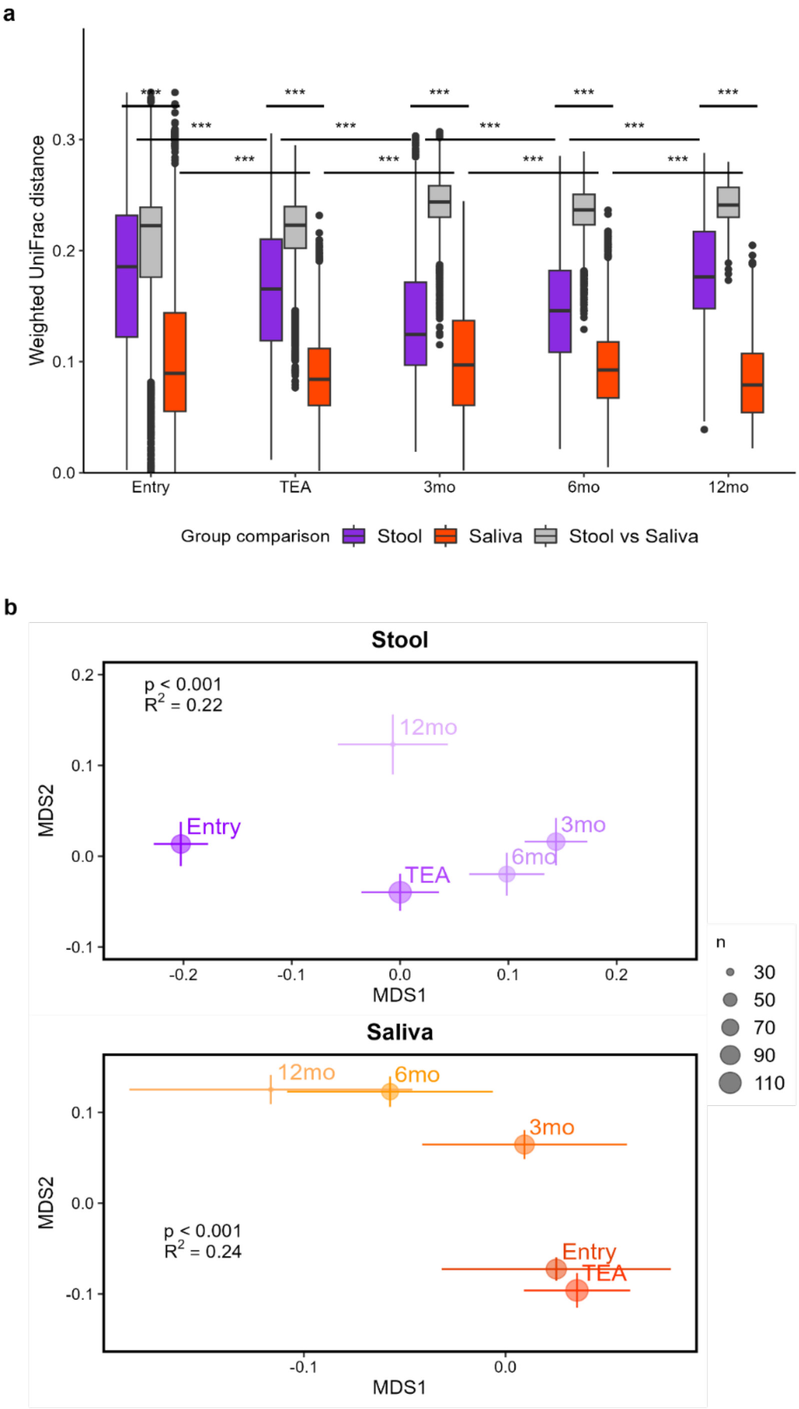

Supplementary Figure 1. **Beta diversity of late and moderate preterm stool and saliva samples over the first year of life**. a) Weighted UniFrac distances are shown for comparisons of stool and saliva within sample types at given time points. Boxes are coloured according to the group comparison and show median (centre line) and interquartile ranges (IQR) (box limits); whiskers extend ±1.5∗IQR from box's quartile. Points outside the whiskers represent outliers. P values are from FDR adjusted Wilcoxon rank sum tests; *** indicates p value < 0.001. b) NMDS plots showing clustering by time point in stool and saliva. Plots are based on ordination of weighted UniFrac distances and show the mean centroids for each group. Centroid size is based on the number (n) of samples and bars represent the ±95% confidence interval. P values and R^2 values on plot faces are from PERMANOVA. TEA, term equivalent age; mo, months corrected age.

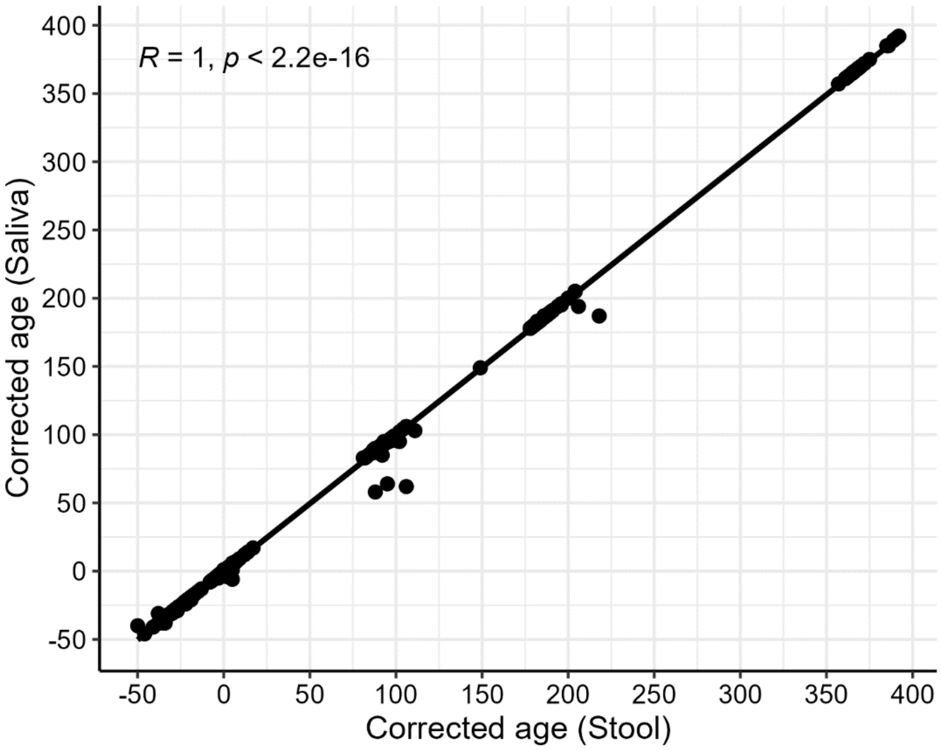

Supplementary Figure 2. **Correlation plot of ages of paired stool and saliva samples.** The axes represent the corrected age of the infant's stool or saliva in days. R value and p value shown are from Spearman's correlation.

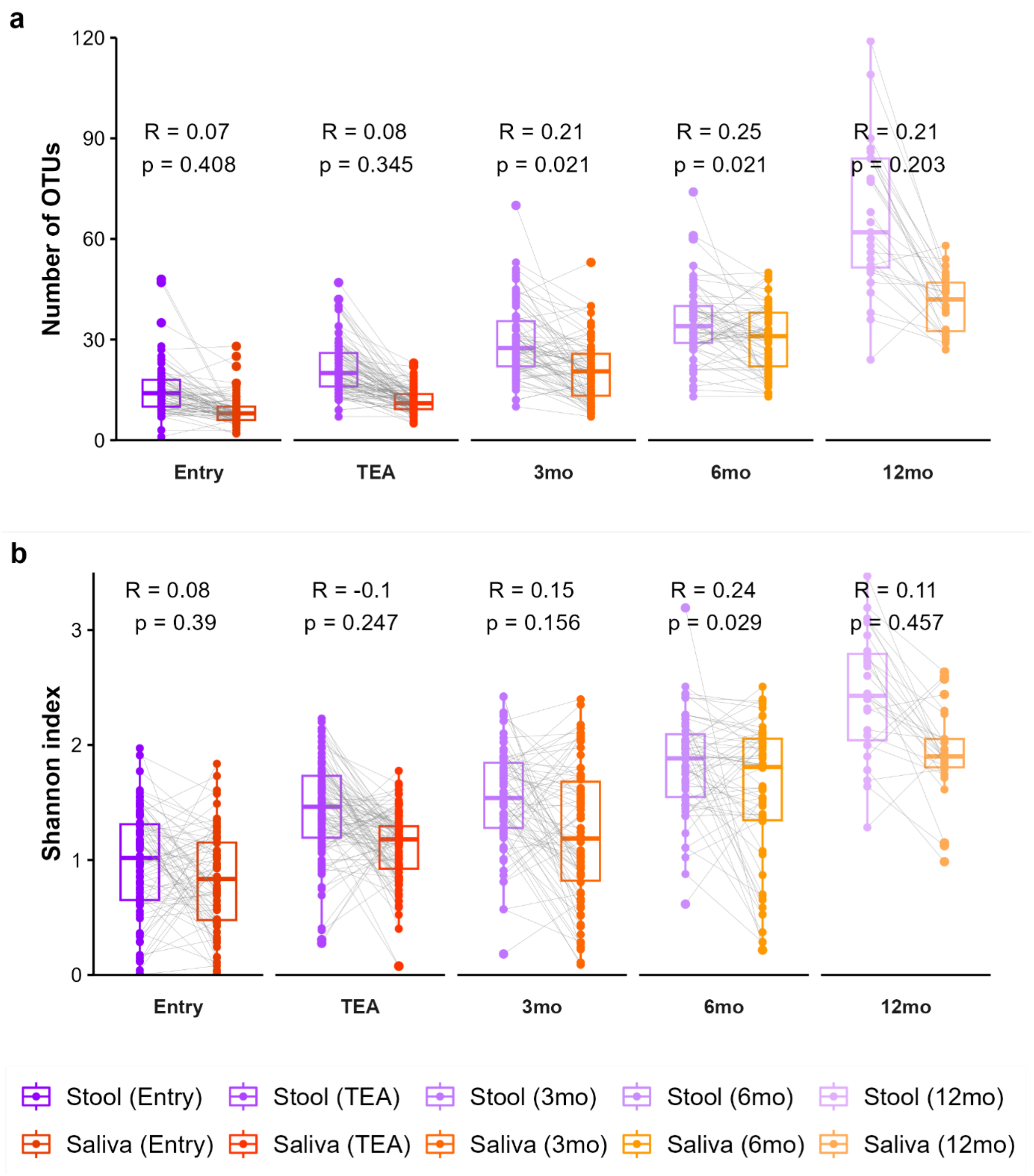

Supplementary Figure 3. **Alpha diversity of paired stool and saliva samples.** Plots show a) the number of OTUs and b) Shannon index of paired stool and saliva samples at given time points (number of patients = 77 at Entry, 98 at TEA, 74 at 3mo, 61 at 6mo and 27 at 12mo). Boxes are coloured according to time point and sample type and show median (centre line) and interquartile ranges (IQR) (box limits); whiskers extend ±1.5∗IQR from box's quartile. Points outside the whiskers represent outliers. Connecting segments join each individual infant's stool and saliva sample. R values and FDR adjusted p values are from Kendall's rank correlation tests. TEA, term equivalent age; mo, months corrected age.

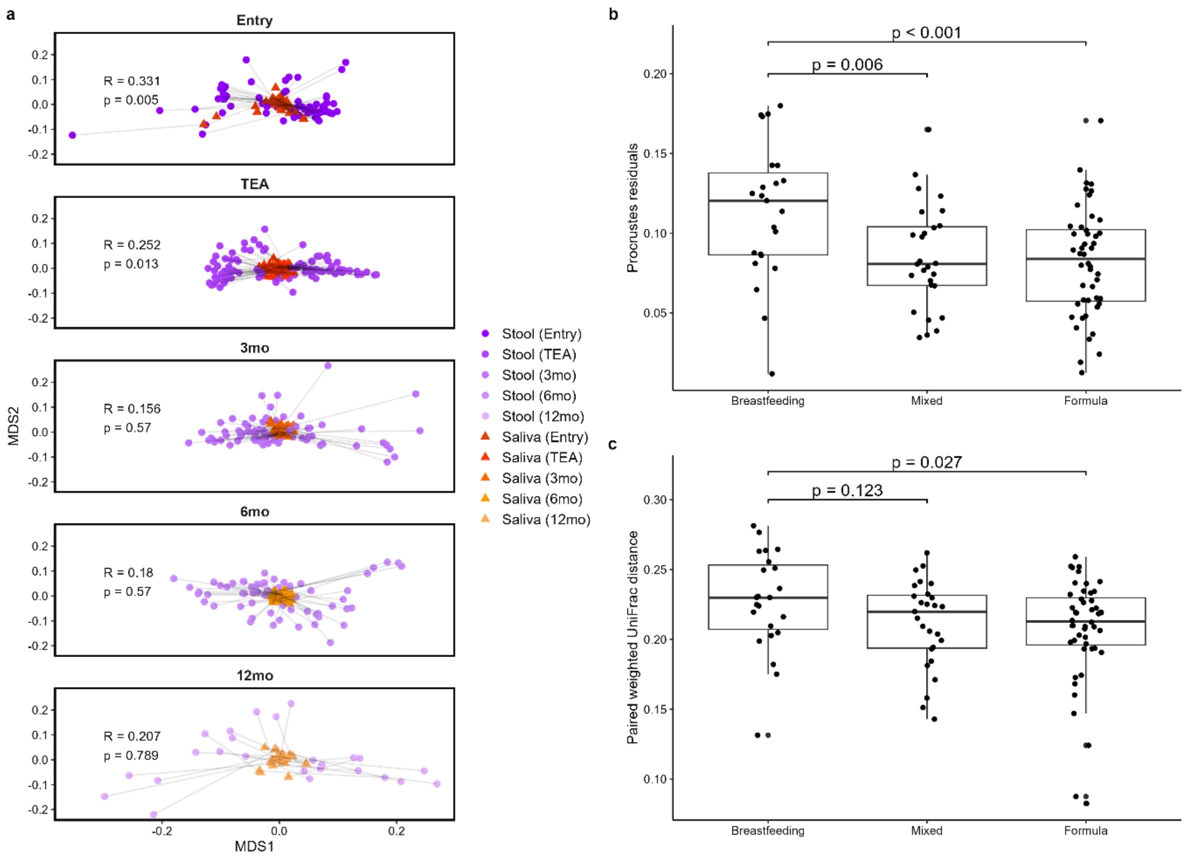

Supplementary Figure 4. **Comparison of matched stool and salivary microbiome profiles within infants.** a) Procrustes analysis showing associations between paired stool and saliva samples. NMDS plots are based on ordination of weighted UniFrac distances. Each point represents a sample and connecting segments join each individual's paired stool and saliva sample. Points are shaped according to sample type and coloured according to sample type and time point. Box plots showing b) residuals from Procrustes analysis and c) weighted UniFrac distances from paired stool and saliva samples according to feeding mode at TEA. Boxes show median (centre line) and interquartile ranges (IQR) (box limits); whiskers extend ±1.5∗IQR from box's quartile. Points outside the whiskers represent outliers. P values are from univariate generalised linear models.

TEA, term equivalent age; mo, months corrected age.

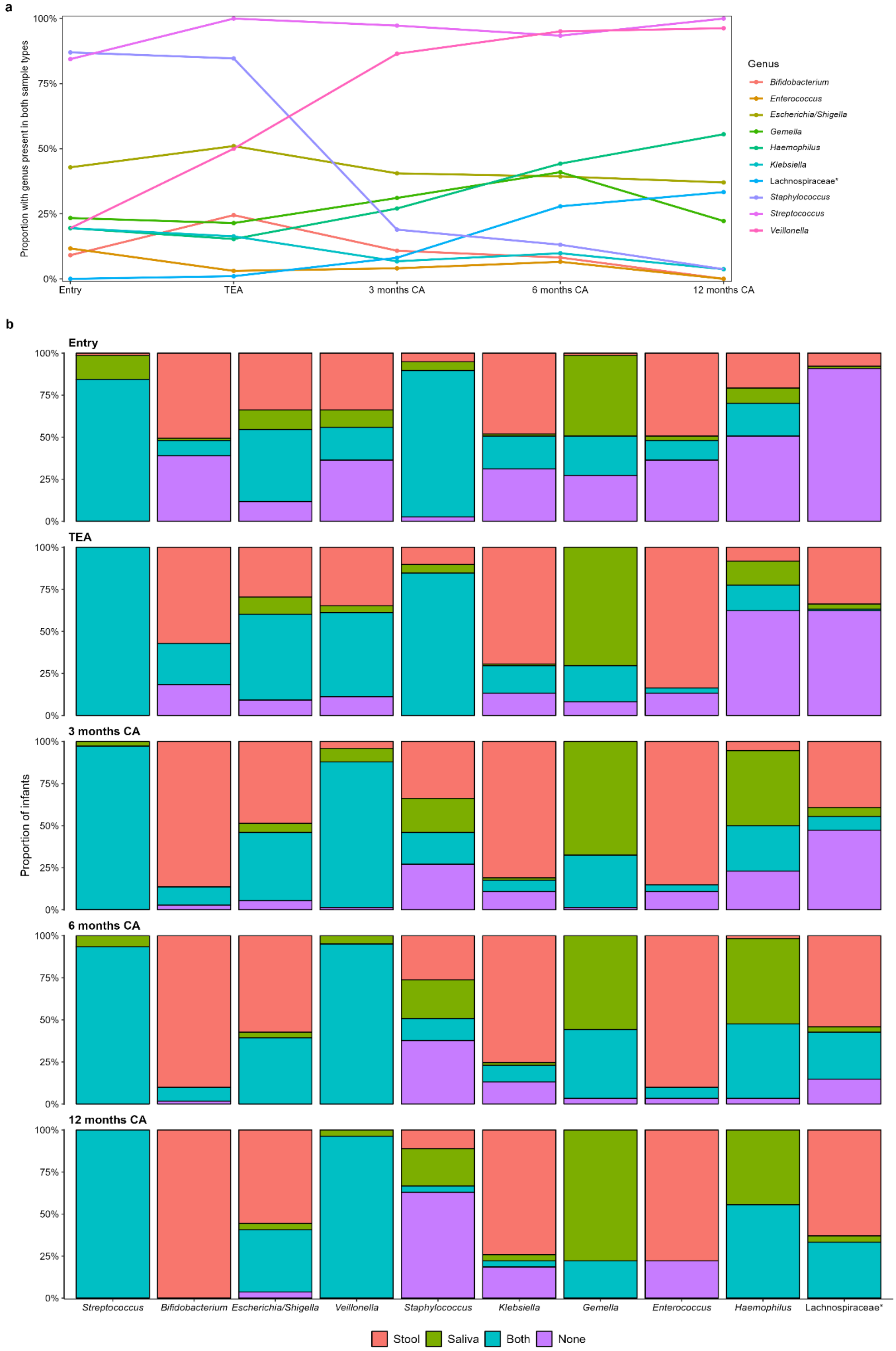

Supplementary Figure 5. Rates of sharing of top ten genera between paired stool and saliva samples. The ten most abundant genera in late and moderate preterm (LMPT) stool and saliva samples are shown at given time points. a) Line plots show the proportion of infants that had each genus detected in both sample types. b) Stacked bar charts are coloured according to the proportion of infants that the genus was detected in stool, saliva, both sample types, or neither sample type. Unclassified genera are represented by their family (*). TEA, term equivalent age; CA, corrected age.

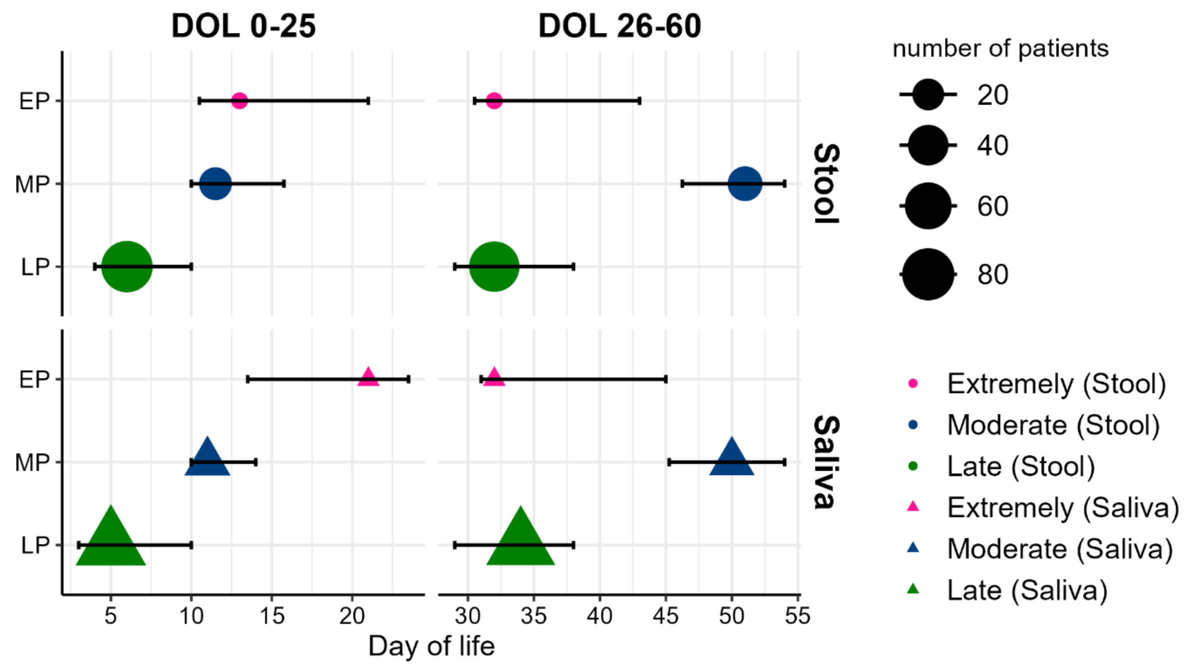

Supplementary Figure 6. Sampling overview of analytical cohort including extremely preterm infants. Day of life (DOL) of patient samples used in the analysis to compare gestational age groups (extremely, moderate, or late preterm; EP, MP, or LP, respectively), separated by time window and sample type (stool or saliva). Points represent the median DOL of samples and bars show the interquartile ranges. Points are coloured according to gestational age group, shaped according to sample type, and sized relative to the number of samples in each group. DOL, day of life; EP, extremely preterm; MP, moderate preterm; LP, late preterm.

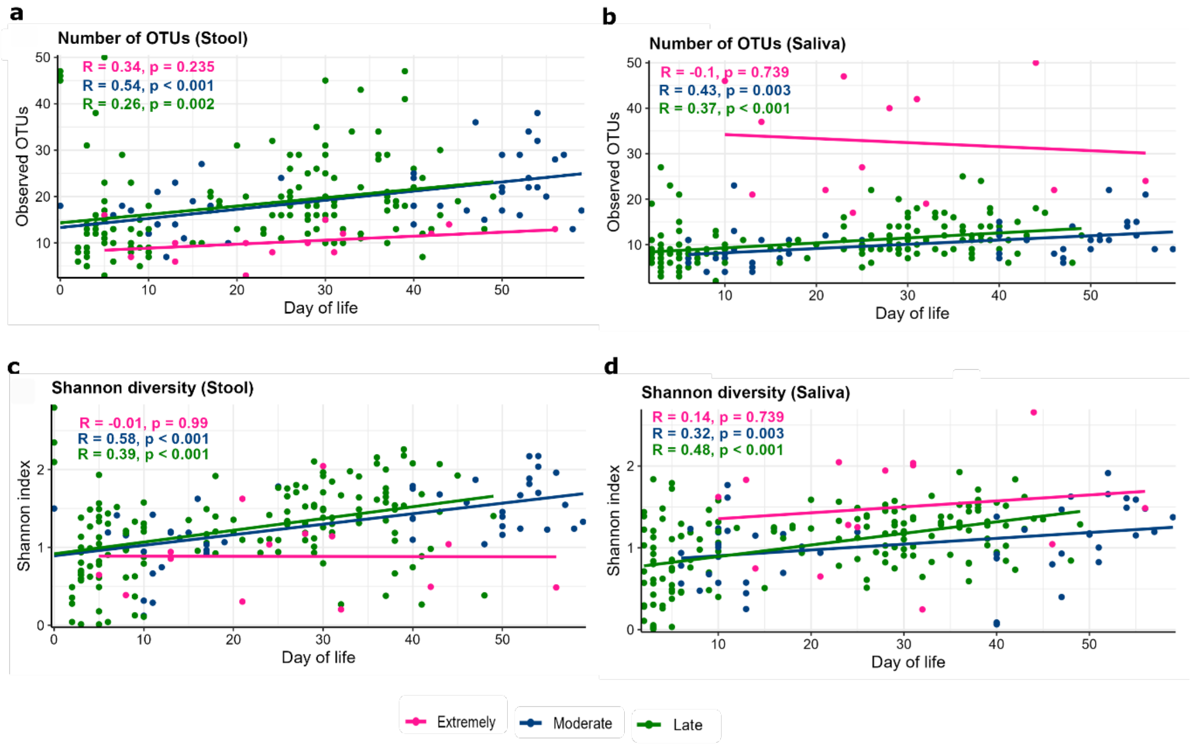

Supplementary Figure 7. Alpha diversity of stool and saliva samples according to gestational age. Correlation plots are shown for a),b) the number of OTUs and c),d) Shannon diversity of stool and saliva samples, respectively. Plots are coloured according to gestational age group (extremely, moderate, or late preterm). R values and p values (FDR corrected) shown are from Pearson's correlations.

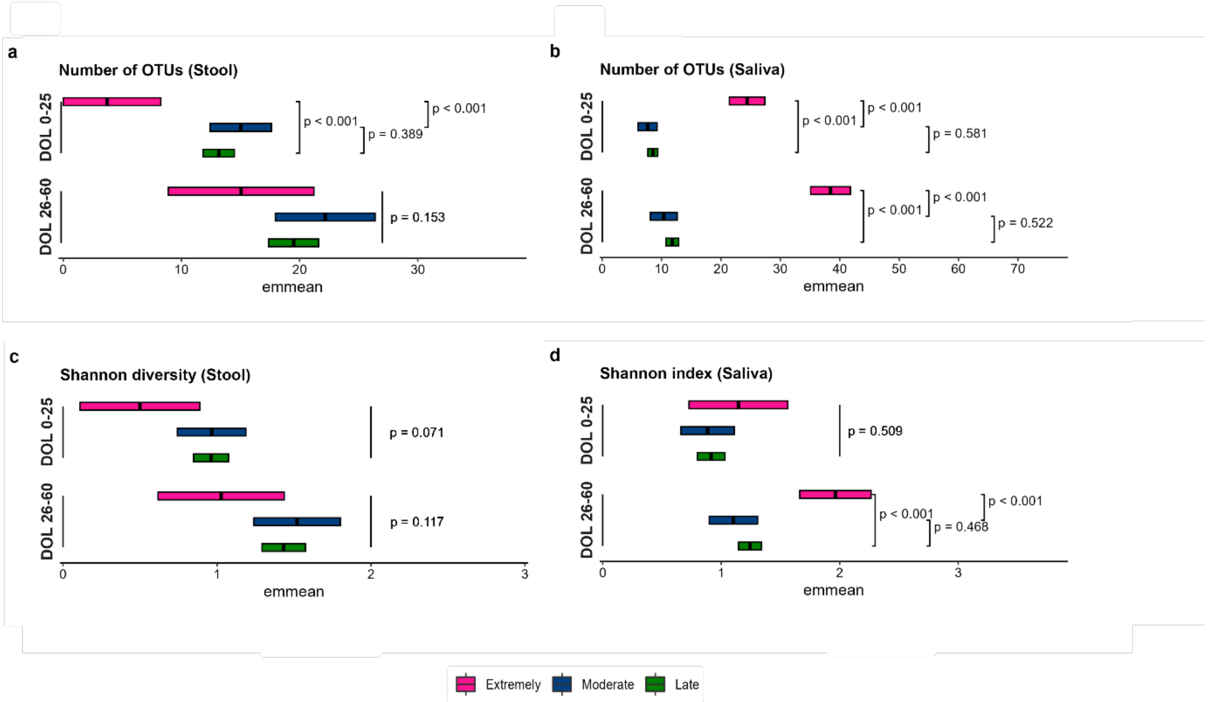

Supplementary Figure 8. Estimated marginal means for alpha diversity based on gestational age. Estimated marginal means (EMMs) (± 95% confidence intervals) are shown for a),b) the number of OTUs and c),d) Shannon diversity of stool and saliva samples, respectively. Plots are facetted by time window and boxes are coloured according to gestational age group (extremely, moderate, or late preterm). EMMs were obtained from robust linear regression models adjusted for sample DOL, delivery mode, season of sample collection, postnatal antibiotics and feeding mode. When gestational age was non-significant ($p > 0.05$) in the global model, p values shown are from Type II Wald Chi-square tests; otherwise ($p < 0.05$), p values are shown for pairwise contrasts after adjustment for multiple comparisons using two-tailed Tukey's HSD method. DOL, day of life.

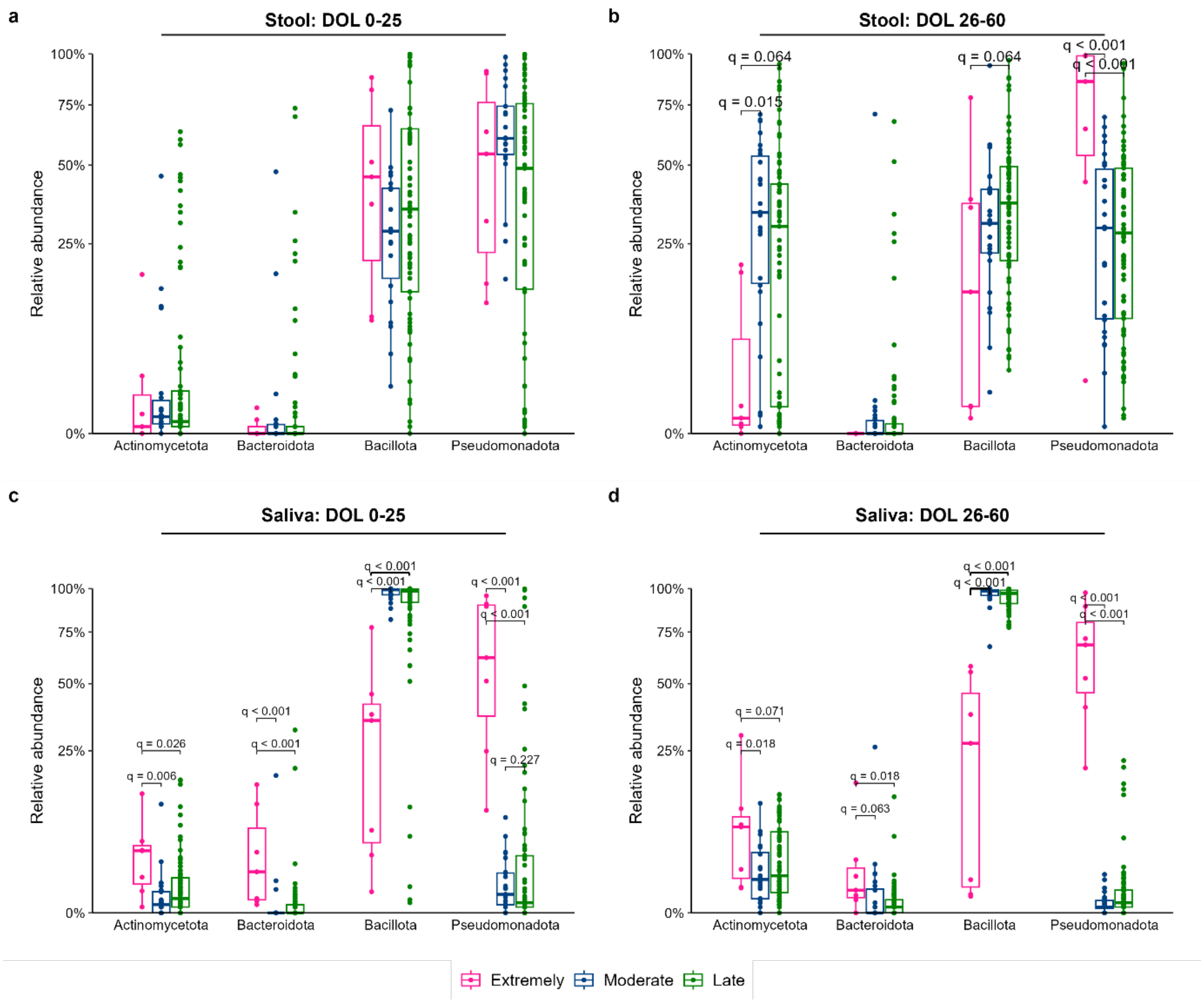

Supplementary Figure 9. Relative abundances of the top four phyla in stool and saliva samples according to gestational age at given time windows. Plots show the relative abundances of phyla in a,b) stool and c,d) saliva samples. Boxes are coloured according to gestational age group (extremely, moderate, or late preterm) and show median (centre line) and interquartile ranges (IQR) (box limits); whiskers extend ±1.5∗IQR from box's quartile. Points outside the whiskers represent outliers. Q values (FDR adjusted p values) are shown for significant ($q < 0.25$) differences between gestational age groups detected by MaAsLin2, after adjusting for sample DOL, delivery mode, season of sample collection, postnatal antibiotics and feeding mode. DOL, day of life.
